# Classification of Parkinsonian Syndromes from FDG-PET Brain Data Using Decision Trees with SSM/PCA Features

**DOI:** 10.1155/2015/136921

**Published:** 2015-03-31

**Authors:** D. Mudali, L. K. Teune, R. J. Renken, K. L. Leenders, J. B. T. M. Roerdink

**Affiliations:** ^1^Johann Bernoulli Institute for Mathematics and Computer Science, University of Groningen, Nijenborgh 9, 9747 AG Groningen, Netherlands; ^2^Department of Neurology, University Medical Center Groningen, University of Groningen, Hanzeplein 1, 9700 RB Groningen, Netherlands; ^3^Neuroimaging Center, University Medical Center Groningen, University of Groningen, Antonius Deusinglaan 2, 9713 AW Groningen, Netherlands

## Abstract

Medical imaging techniques like fluorodeoxyglucose positron emission tomography (FDG-PET) have been used to aid in the differential diagnosis of neurodegenerative brain diseases. In this study, the objective is to classify FDG-PET brain scans of subjects with Parkinsonian syndromes (Parkinson's disease, multiple system atrophy, and progressive supranuclear palsy) compared to healthy controls. The scaled subprofile model/principal component analysis (SSM/PCA) method was applied to FDG-PET brain image data to obtain covariance patterns and corresponding subject scores. The latter were used as features for supervised classification by the C4.5 decision tree method. Leave-one-out cross validation was applied to determine classifier performance. We carried out a comparison with other types of classifiers. The big advantage of decision tree classification is that the results are easy to understand by humans. A visual representation of decision trees strongly supports the interpretation process, which is very important in the context of medical diagnosis. Further improvements are suggested based on enlarging the number of the training data, enhancing the decision tree method by bagging, and adding additional features based on (f)MRI data.

## 1. Introduction

Neurodegenerative brain diseases like Parkinson's disease (PD), multiple system atrophy (MSA), or progressive supranuclear palsy (PSP) are difficult to diagnose at early disease stages [1]. It is important to develop neuroimaging techniques that can differentiate between the various forms of Parkinsonian syndromes and stages in progression. Early disease detection is aided by brain imaging techniques like [18F]-fluorodeoxyglucose (FDG) positron emission tomography (PET) and magnetic resonance imaging (MRI) to obtain image data and derive significant patterns of changed brain activity. Several techniques have been developed to identify disease-related network patterns of cerebral glucose metabolism.

Covariance techniques like principal component analysis (PCA) can be used to extract significant patterns from brain image data. PCA is known for its capability to identify patterns in high-dimensional data like brain image data. A possible approach to biomarker identification is the scaled subprofile model/principal component analysis (SSM/PCA) method [2, 3]. SSM/PCA is a feature extraction method which enhances identification of significant patterns in multivariate imaging data. This method has been extensively applied to positron emission tomography data to identify brain patterns which display significant differences between healthy controls and Parkinsonian conditions. The SSM/PCA method helps to reduce data dimensions and to reveal the brain patterns characteristic for a certain Parkinsonian syndrome. Resting state metabolic networks obtained from FDG-PET scans were used to identify disease-related metabolic brain patterns of PD, MSA, and PSP [4–7]. In a previous study by Tang et al. [8], it was demonstrated that by using an image-based classification routine, it was possible to distinguish with high specificity between PD and MSA/PSP and in a second step between MSA and PSP as compared to controls.

In a recent study by Hellwig et al. [9], the diagnostic accuracy of FDG-PET in discriminating Parkinsonian patients was investigated. FDG-PET scans were analyzed by visual assessment including individual voxel based statistical maps (a 3D stereotactic surface projection technique; 3D-SSP). These studies compared only two classes at a time or on two levels (healthy and patient group, or two patient groups). This puts forward a research challenge to improve the SSM/PCA method to be able to distinguish different neurodegenerative brain diseases from each other in one analysis.

For this reason we consider machine learning approaches like decision tree methods to be able to compare more than two patient groups at the same time and possibly detect subtypes within patient groups. The C4.5 decision tree classification algorithm by Quinlan [10] is used to classify Parkinsonian conditions from FDG-PET imaging data. This algorithm uses a feature selection criterion known as information gain to induce decision trees from training data. The subject scores derived from the SSM/PCA method are used as input features for the C4.5 algorithm. After the training phase, the decision trees can then be used as predictors for unseen cases with unknown disease type. Decision trees are known to be intuitive and easily understandable by humans [11]. In other words, they can be easily visualized and interpreted by the clinicians.

In this paper, we combine the SSM/PCA method in a novel way with the C4.5 decision tree classification algorithm which classifies Parkinsonian disorders according to their respective disease types. We also compare the decision tree method with a number of other classifiers with respect to different criteria, such as performance and interpretability by humans.

## 2. Materials and Methods

**Figure 1:**
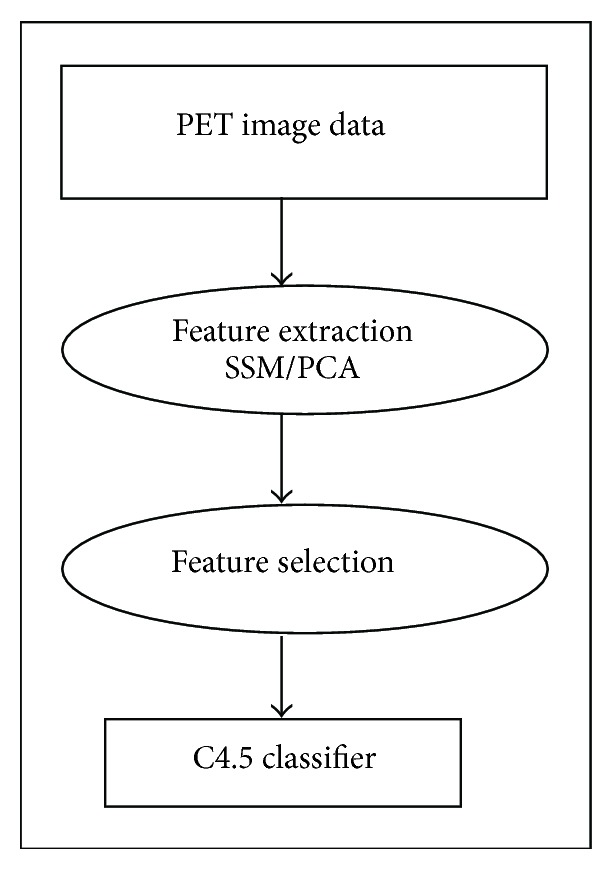
Classification steps.

The extraction of patterns and classification involves four main steps: data acquisition, feature extraction, feature selection, and classification (see Figure 1).

### 2.1. Data Acquisition

FDG-PET scans selected from a previous study [12] describing 18 healthy controls (HC), 20 PD, 21 MSA, and 17 PSP patients were included for the present analysis. At the time of referral for imaging, the clinical diagnosis of most patients was uncertain. The final clinical diagnoses according to established clinical research criteria [1, 13, 14] were made after a follow-up time after scanning of 4 ± 3 years (y) in PD, 2 ± 1 y in MSA, and 3 ± 2 y in PSP. Included PD patients were 9 male (M) and 11 female (F), 6 right body side affected and 14 left side affected, with mean age of 63 ± 9 y and Disease Duration (DD) at scanning of 3 ± 2 years. Fourteen probable MSA and 7 possible MSA patients (10 M and 11 F, age 64 ± 10 y; DD 4 ± 2 y) and 13 probable and 4 possible PSP patients (9 M and 8 F, age 68 ± 8 y; DD 2 ± 1 y) were included.

### 2.2. Feature Extraction

We reimplemented the SSM/PCA method in MATLAB based on the description by Spetsieris and Eidelberg [6, 15–17]. First, the FDG-PET images are loaded in a data matrix *P*_*sv*_, and a mask is applied to each subject image in *P*_*sv*_ (*s*[1,…, *M*] refers to subjects and the column index *v* refers to voxels) to remove all voxels with intensity value less than 35% of the whole brain volume maximum. Then the subject matrix is log-transformed and doubly centered to create a subject residual profile (SRP) matrix SRP_*sv*_. PCA is then applied to the matrix SRP_*sv*_ to obtain its eigenvectors. These eigenvectors are called Group-Invariant Subprofile (GIS) patterns (GIS_*k*_, *k* = 1,2,…, *M*) and represent characteristic disease-related brain patterns. Furthermore, subject scores are computed as the contribution of each subject image to a disease-related pattern GIS_*k*_.

The SSM/PCA method was applied to several data groups (disease group(s) compared to healthy controls) in training set(s) from which disease-related patterns (GIS_*k*_) were extracted with positive and negative loadings (voxel weights) [18]. The brain images from the training set are weighted onto the patterns to obtain subject scores, which depict how much each subject image contributes to a pattern.

*Subject Scores as Features for Classification.* Features are usually derived as characteristics of an object such as texture, color, or shape [19], which can be computed for each subject (data set) separately. The use of PCA-based subject scores as features deviates significantly from the standard situation through the fact that features now depend on the whole dataset. Also, the number of features is, at least initially, equal to the number of data sets. So when a subject is removed or added to the data collection the scores of all the other subjects change as well. Therefore, there is a need to redo the SSM/PCA procedure once the dataset changes to obtain new scores.

### 2.3. Decision Tree Classification

The C4.5 decision tree method [20] is a supervised learning strategy which builds a classifier from a set of training samples with a list of features (or attributes) and a class label. The algorithm splits a set of training samples into subsets such that the data in each of the descending subsets are “purer” than the data in the parent subset (based on the concept of information gain from information theory). Each split is based on an optimal threshold value of a single feature. The result is a tree in which each leaf carries a class name and each interior node specifies a test on a particular feature. The tree constructed in the training phase of a decision tree classifier can be drawn easily to understand graphical representation which shows the successive features and threshold values which the algorithm has used to separate the data set in nonoverlapping classes. Once a tree has been obtained from the training samples, it can be used for testing to classify unseen cases where the class label is unknown.

The C4.5 decision tree algorithm [10] has been used in many previous studies, ranging from diatom identification [21] to classification of anomalous and normal activities in a computer network to curb intrusions [22]. The method has also been applied to improve accuracy in multiclass classification problems. For example, Polat and Güneş [23] applied a novel hybrid classification system based on the C4.5 decision tree classifier and one-against-all approach, obtaining promising results. In addition, Ture et al. [24] analysed several decision tree methods (CHAID, CART, QUEST, C4.5, and ID3) together with Kaplan-Meier estimates to investigate their predictive power of recurrence-free survival in breast cancer patients. In summary, decision trees are considered to be powerful for classification and are easy to interpret by humans. Not only are they simple and effective but also they work well with large datasets [25].

*Decision Tree Classification of Parkinsonian Syndromes.* Using the C4.5 machine learning algorithm, we trained classifiers on subject scores of extracted patterns for healthy subjects and subjects with known types of neurodegenerative disease. The result is a pruned decision tree showing classified subject images. The goal of pruning is to obtain a tree that does not overfit cases. Note that it would be possible to obtain 100% correct classification in the training phase by using a less stringent pruning strategy. However, this would come at the expense of generalization power on unseen cases.

**Figure 2:**
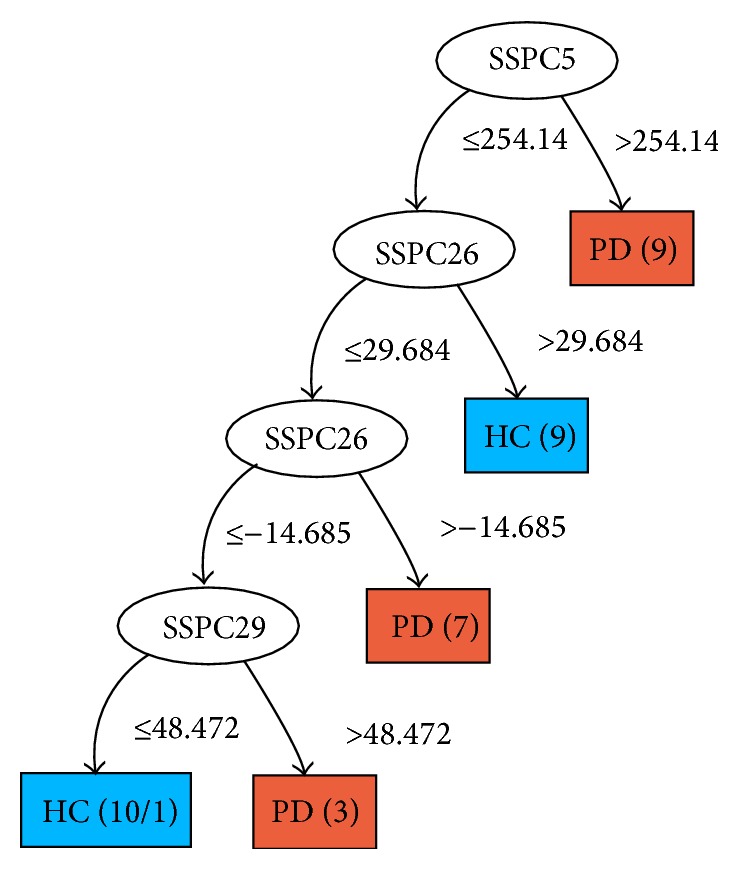
The decision tree built from the PD-HC dataset. Oval-shaped interior nodes: features (subject scores) used to split the data. Threshold values are shown next to the arrows. Rectangular leaf nodes: the final class labels (red = PD, blue = HC).

In contrast to applications of the SSM/PCA method which make a preselection of principal components (GIS vectors) on which the classification will be based, the C4.5 algorithm uses all principal components and the corresponding subject scores as input. The algorithm itself determines which principal components are most discriminative to separate the data set into classes. More discriminative components appear higher in the decision tree, that is, closer to the root; refer to Figure 2 for an example, where the subject score SSPC5 is the most discriminative feature.

In order to apply the C4.5 classifier to unseen cases, the required subject scores for testing are first computed by projecting the SRP of the new subject on the GIS profiles of the training set. The computation of the SRP for the unseen case involves centering along the subject dimension, that is, subtracting the GMP (group mean profile). The assumption is that this GMP can be obtained from the reference group only, that is, the group used for training the classifier; see the discussion in Spetsieris et al. [17, page 1244].

### 2.4. Other Classifiers

We also applied a number of other classifiers: nearest neighbors; linear classifiers: linear discriminant analysis and support vector machines; random forests, which is an extension of decision trees; classification and regression trees (CART) for predicting real/continuous variables; and naive Bayes, a probabilistic classifier. Linear classifiers in particular are simple to implement. They are known to work better in situations where the data is uniformly distributed with equal covariance.

*Nearest Neighbors (NN).* NN is a classification method which assigns a class to a new data point based on the class of the nearest training data point(s). In the *K*-NN (*K*-Nearest Neighbors) method, distances to the neighbors are computed first. Then, a new data point receives the majority label of the *K* nearest data points. 

*Linear Discriminant Analysis (LDA).* LDA, like PCA, is used for data classification and dimensionality reduction. This classifier maximizes the between-class variance and minimizes the within-class variance to ensure a clear separation in datasets. Accordingly, the training data are first transformed; then the data in the transformed space are classified as belonging to a class which minimizes the Euclidean distance of its mean to the transformed data [26]. 

*Support Vector Machine (SVM).* SVM performs classification by generating an optimal decision boundary in the form of a hyperplane which separates different classes of data points in the feature space. The decision boundary should maximize the distance between the hyperplane and support vectors called the margin [27]. 

*Random Forests.* Random forests is a machine learning method for classification of objects based on a majority vote of a multitude of decision trees. This method combines bagging (random selection of cases) and random selection of features (at each node) during the training phase. Also, the trees are not pruned. 

*Classification and Regression Trees (CART).* CART, just like C4.5, is a decision tree learning method. However, in addition to using decision trees as predictors, CART includes regression trees for predicting continuous variables. 

*Naive Bayes.* This is a method that classifies data points based on their likelihood and the prior probabilities of occurrences of known classes. The final classification is achieved by combining the prior and the likelihood to form a posterior probability using Bayes' rule. Overall, the new data will belong to a class which maximizes the posterior probability.

## 3. Results and Discussion

### 3.1. Results for Decision Tree Classifiers

Decision tree classifiers were trained by applying the C4.5 algorithm to individual (each disease group versus healthy controls) and combined datasets of PD, PSP, and MSA patients and healthy controls (HC) with known class labels, as listed in Section 2.1. For the individual datasets, we were interested in identifying features which best separate two groups (i.e., a disease group from healthy controls). For the combined datasets we compared all the groups, that is, PD, MSA, PSP, and HC to each other to obtain feature(s) which can separate the four groups. Tree pruning was carried out by using the default values of the C4.5 algorithm [10].

#### 3.1.1. Building Classifiers for Individual Datasets

Decision tree classifiers were built in the training phase from the individual datasets (PD, PSP, and MSA) compared to the HC group of 18 subjects.

*PD Group.* The decision tree built from the PD-HC dataset (18 healthy and 20 PD subjects) is illustrated in Figure 2. The subject scores derived from 38 principal components (GIS vectors) are the attributes on which decisions are made. They are represented as oval-shaped interior nodes in the tree. Next to the arrows the threshold values that were used to split the dataset are shown. Likewise, the leaf nodes, represented as rectangles, show the final class or decision made at that level of the tree (e.g., PD or HC in Figure 2). Red and blue colors are used to indicate cases labeled as PD and healthy, respectively. The numbers between brackets in the rectangles show the total number of cases classified at that leaf. Additionally, the number after the slash (if present) represents the number of misclassified cases at that leaf.

As can be seen in Figure 2, the classifier chooses the subject score based on component 5 (SSPC5) to make the first split. In the right subtree, nine PD subjects > 254.14 are identified. The classifier goes on to test the rest of the subjects based on component 26, where nine subjects (subject score > 29.684) are identified as HC, and so forth. Only one PD subject is misclassified as HC, as can be seen in Figure 2 in the lower left rectangle.

**Figure 3:**
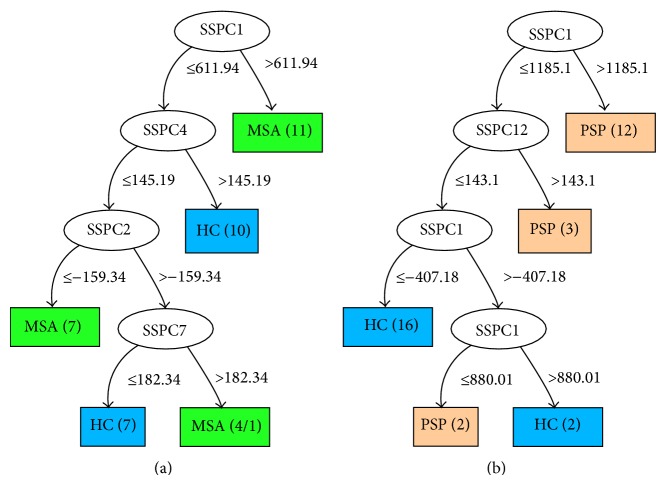
The decision trees built from the MSA-HC (a) and PSP-HC (b) datasets. For details, refer to Figure 2.

*MSA Group.* The decision tree built from the MSA-HC dataset (18 healthy and 21 MSA subjects) is illustrated in Figure 3(a). The attributes are subject scores derived from 39 principal components. Again, one HC subject is misclassified.

*PSP Group.* The decision tree built from the PSP-HC dataset (18 healthy and 17 PSP subjects) is illustrated in Figure 3(b). The attributes are subject scores derived from 35 principal components.

#### 3.1.2. Building Classifiers on Combined Datasets

**Figure 4:**
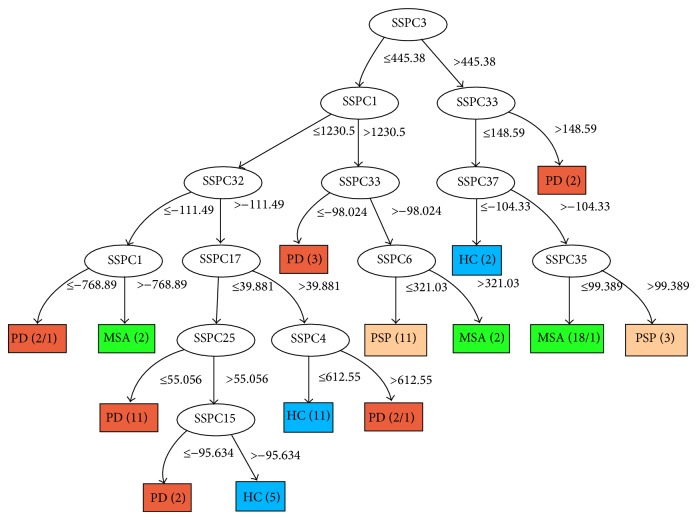
The decision tree built from the combined PD-PSP-MSA-HC dataset.

We also applied the C4.5 classification algorithm to the combined datasets consisting of all four groups. Therefore, the dataset consisted of 76 subjects, 18 HC, 20 PD, 21 MSA, and 17 PSP. Subject scores were obtained by applying the SSM/PCA method to the combined group. The resulting decision tree is shown in Figure 4. Three PSP subjects are classified erroneously, two as PD and one as MSA.

#### 3.1.3. Leave-One-Out Cross Validation

In leave-one-out cross validation (LOOCV), a single observation from the original dataset is used as the validation set (also known as test set) and the remaining observations form the training set. This procedure is repeated *N* times where each observation is used once as a validation set.

The LOOCV method was applied to individual and combined datasets, that is, PD-HC, MSA-HC, PSP-HC, and the combined dataset PD-MSA-PSP-HC to estimate classifier performance on unseen cases. Here performance is defined as the percentage of correct classifications over the *N* repetitions. To ensure that attributes of the training set, and thus the trained classifier, are independent of the validation sample, the test subject was removed from the initial dataset before applying the SSM/PCA method to the training set (with *N* − 1 samples) for obtaining the subject scores needed to train the C4.5 decision tree classifier. The classifier was then used to determine the label for the test subject. This procedure was applied for each of the *N* subjects in the original dataset.  Table 1 shows the classifier performance.

As seen in Table 1, the C4.5 classifier performs highest with the PSP group at 80% and lowest with the PD group at 47.4%. The feature at the root of a decision tree is most significant in classification, since it has the highest information gain (see Section 2.3). As seen in Figure 3, feature 1 (i.e., the subject score on principal component 1) is chosen by the classifier in making a first separation between healthy and PSP/MSA subjects. Moreover, we observed that for the PSP-HC group feature 1 occurs as the root for all LOOCV trees. This behaviour is strongly linked to the high performance of the PSP group, since the classifier is utilizing the relevant feature(s) for the separation of the groups.

The MSA-HC dataset has the second best performance and we observed that the feature at the root of the MSA-HC tree in Figure 3(a) also appears as root in 32 out of 39 trees in LOOCV. On the contrary, for the PD group, different features were chosen by the classifier as root nodes of the different LOOCV trees. Apparently, the different features contain only weakly relevant information to separate the healthy group from the PD group. In this case, application of the decision tree method with all features included leads to a form of overfitting. We attribute this to the fact that the PD group is quite similar to the HC group, at least with respect to the features we have measured. The early PD group might contain other disease subtypes which need to be identified.

For the combined dataset (see Figure 4), feature 3 occurs as the root node, so it is the best at separating the four groups (HC, PD, MSA, and PSP). Furthermore, the same feature occurs as the root node in 63 out of 76 LOOCV trees, implying consistency of the classifier. However, the performance for the combined group is low, that is, 53.9% (the number of correctly classified healthy controls, PD, PSP, and MSA subjects is equal to 55.6%, 35%, 58.5%, and 66.7%, resp.). Our explanation is that the number of subjects per class is quite low given the large variability in each group. In addition, the combined group is not well balanced in view of a relatively small size of the healthy subject group versus the combination of the three disease groups.

*Permutation Test.* In order to determine the significance of the performance results we ran a permutation test on the PD-HC, MSA-HC, and PSP-HC groups [28, 29]. The steps of the procedure are as follows:

1. for each group, perform a LOOCV on the original subject labels to obtain a performance *P*_*O*_;
2. repeatedly permute the labels and then do a LOOCV to obtain performances *P*_*i*_ for *i* = 1,…, *N*_perm_ (we used *N*_perm_ = 100);
3. compute the *p* value as the total number of all *P*_*i*_ greater or equal to *P*_*O*_, divided by *N*_perm_.

If *p* < 0.05, the original LOOCV result is considered to be statistically significant.

The results of the permutation test were as follows. For the PSP-HC group, *p* = 0.00; for the MSA-HC group, *p* = 0.01; for the PD-HC group, *p* = 0.62. So we can conclude that for the PSP-HC and MSA-HC groups the performance results are significant. However, for the PD-HC group this is not the case. This is consistent with the lack of robustness of the LOOCV trees we already noted above. The healthy and PD groups are very similar and hard to separate, given the small number of datasets.

#### 3.1.4. Preselection of Features

In the hope to improve the classifier performance, we varied the number of features used to build the classifier in the LOOCV. This was done in two different ways: (i) by choosing the subject scores of the *n* best principal components according to the Akaike Information Criterion (AIC) [30] and (ii) by choosing the first *n* principal components arranged in order of highest to lowest amount of variance accounted for. The classifier performance at the varying numbers of features is shown in Table 2.

As shown in Table 2, the performance of the PD group improves from 47.4% to 63.2% when the number of features is reduced from 100% to 70% and 5%. Also the performance improves when only one best feature according to AIC is used to build the classifier. Likewise the performance of the MSA and PSP groups improves from 71.8% to 74.4% and 80% to 82.9%, respectively, when the number of features is reduced. Notable is that the number of features at which distinct groups perform best may differ. Specifically, when using the AIC for preselection, not always one feature is good enough to separate groups. This can be seen for the MSA group where five best features were required to obtain the best performance. Overall, preselection/reduction of features to include relevant features can boost classifier performance.

#### 3.1.5. Disease Groups versus Each Other

Disease groups were compared to each other in a binary classification, that is to say, the PD group of 20 subjects versus the MSA group of 21 subjects, PD group of 20 versus PSP group of 17, and MSA group of 20 versus PSP group of 17.

As seen in Table 3, PD versus MSA has the highest performance with a relatively high sensitivity and specificity; consequently PD can be separated rather well from MSA. For the PD versus PSP and MSA versus PSP groups the performance is slightly lower. The performance of all groups slightly increases when features are reduced to only 5 according to AIC. In spite of the high performance of the PSP group versus the healthy group as seen in Table 1, PSP performs relatively low when compared to the other disease groups (PD and MSA). Apparently, the PSP features look more like those of PD or MSA patients than those of healthy controls.

#### 3.1.6. Combined Disease Groups

**Figure 5:**
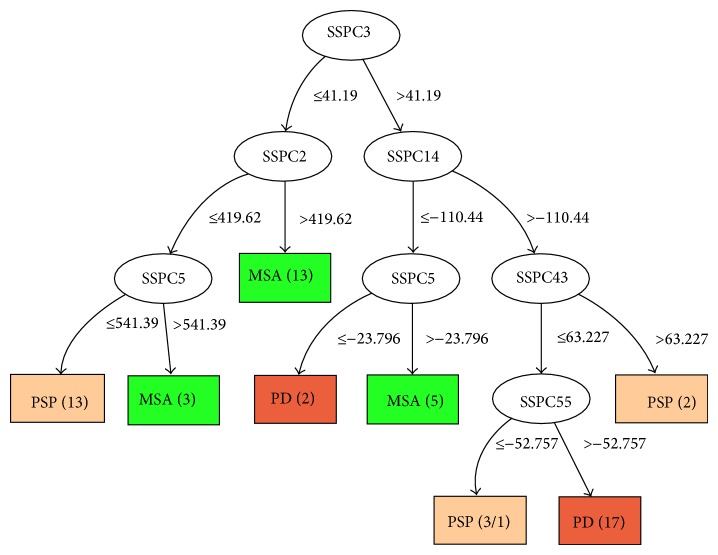
The decision tree built from the disease groups compared to each other, that is, PD-PSP-MSA dataset.

Our main interest is to distinguish the Parkinsonian syndromes from each other. Therefore, we combined all disease groups (i.e., PD, PSP, and MSA) without the healthy controls in a decision tree multiclassification and applied LOOCV (at 100% features used). The performance of the classifier is 65.5%, with 75% correctly classified PD subjects, 47.1% correctly classified PSP subjects, and 71.4% correctly classified MSA subjects. Altogether the PSP group has the lowest number of correctly classified subjects, which is in agreement with the previous observation that it has similarities to PD and MSA.  Figure 5 shows the decision tree diagram obtained after training the classifier with all features. Only one PD subject is misclassified as PSP.

*Varying the Number of Features for Classification.* Several LOOCV experiments were carried out while varying the number of features used to build the classifier. The highest performance was achieved when including 25% of all features. Results for 100, 50, and 25% of all features are shown in Table 4.

### 3.2. Results for Other Classifiers

We used “scikit-learn” [31], a software package that includes a variety of machine learning algorithms, to obtain classification results for a number of other classifiers. The classifiers used were described in Section 2.4. In principle, we should test on subject scores obtained from the leave-one-out method before applying the SSM/PCA method. However, this would lead to a very time-consuming procedure. Since our goal is to obtain an impression of the improvements possible by using other classifiers, we instead applied LOOCV on subject scores obtained from applying the SSM/PCA method to the whole training set (all subjects included).

Performances of PD, MSA, and PSP groups versus healthy controls are shown in Table 5. No preselection of features was applied.

### 3.3. Discussion

The LOOCV performance shown in Table 5 is highest for the SVM and NN classifiers. These classifiers perform better than C4.5, especially for the PD-HC group. We attribute this to the fact that SVM and NN only have one decision boundary. On the other hand, C4.5 has several decision boundaries, one for each internal node of the decision tree. Thus a subject is tested more than once and may become vulnerable to misclassification in the case where the features depict noise or are irrelevant. CART is quite similar to C4.5; for the PD and PSP groups it has a higher performance but for MSA it is considerably lower.

Decision tree methods are faced with the problem of overfitting, which causes all training cases to be correctly classified but with limited generalizability; that is, the learned tree tends to be so perfect that it is prone to misclassify unseen cases. Also, providing many features to the decision tree inducer can cause a low performance due to irrelevant and redundant features, especially when the number of subjects is relatively small. Moreover it has been observed that C4.5's feature selection strategy is not optimal, so having irrelevant and correlated features can degrade the performance of the classifier [25]. In addition, the C4.5 classifier has been reported to perform lower when it comes to continuous attributes, which is the case in our study (as subject scores are continuous) [32]. However, with preselection of features and pruning decision trees after construction, these problems can be reduced. Indeed, we found an increase in performance, especially for the PD-HC group (see Table 2).

When the number of subjects in the training set is large enough, the decision tree classifier will be capable of performing subtype classification of Parkinsonian syndromes. Another important advantage of the decision tree method over most other methods is that it provides an intuitive way to get insight in the behavior of the classification algorithm to physicians. Drawings of decision trees are human understandable, and the way a decision tree algorithm takes repeated decisions with respect to multiple criteria is close to the way humans carry out multicriteria decision making. Likewise, the significance of a particular feature is recognizable from the level in which the corresponding node appears in the constructed tree. Therefore, we have the opportunity to use human intelligence in the decision tree method to select those features (i.e., the corresponding disease-related patterns) that best distinguish between healthy subjects and patients.

## 4. Conclusions

Using the SSM/PCA method, Group-Invariant Subprofile (GIS) patterns were extracted from FDG-PET data of patients with three distinct groups of syndromes, that is, Parkinson's disease (PD), multiple system atrophy (MSA), and progressive supranuclear palsy (PSP), always matched with a healthy control (HC) group. The subject scores corresponding to these patterns served as the feature set for the C4.5 decision tree classification algorithm. Classifiers were constructed for future prediction of unseen subject images. Validation of classifiers to ensure optimal results was performed using the leave-one-out cross validation (LOOCV) method. A permutation test was performed to assess the statistical significance of the results.

We also compared the C4.5 classifier to various other classification algorithms, that is, nearest neighbors, linear SVM, random forest, naive Bayes, LDA, and CART. Of all classifiers, the performance of nearest neighbors and linear SVM was highest. We found that most classifiers perform relatively well for the PSP-HC and MSA-HC groups but less well for the PD-HC group. This may be closely linked to the fact that the FDG-PET activation pattern of (early stage) PD patients is close to that of normal subjects, whereas there is one distinctive feature which is present in MSA (low uptake in putamen) and PSP (low frontal uptake), respectively, and absent in controls.

In clinical practice, the main problem is not so much to distinguish patients with Parkinsonian syndromes from healthy controls but to distinguish between the different Parkinsonian diease types. For this reason, we also compared disease groups to each other in a binary classification with promising results: in this case classifier performance was significantly higher also when the PD group was involved. In a recent study, Garraux et al. [33] used Relevance Vector Machine (RVM) to classify 120 Parkinsonian patients on the basis of either binary classification (a single class of 3 atypical Parkinsonian syndromes (APS) versus PD) or multiple classification (PD and the 3 APS separately versus each other). The performance achieved in the study of Garraux et al. was higher than in ours. Note, however, that they had a larger dataset and incorporated bootstrap aggregation (bagging) to boost the performance. We plan to incorporate bagging in future work to improve classifier performance.

To achieve high-quality biomarker identification, one needs to accumulate large numbers of patient data in several phases of disease progression. This is what we are currently pursuing in the GLIMPS project [34] which aims at establishing a national database of FDG-PET scans in Netherlands. Additionally, data could be generated from other imaging modalities such as (f)MRI, ASL, and DTI to enable the collection of a broad set of brain features needed for distinguishing the different disease types.

## Figures and Tables

**Table 1:** Classifier performance for the different data sets (patients versus healthy controls, number of cases in brackets) in the LOOCV, without feature preselection. The column Perf. (%) indicates the percentage of subject cases correctly classified per group, Sensitivity (%) indicates the percentage of correctly classified healthy controls, and Specificity (%) indicates the percentage of correctly classified patients.

Feature set (size)	Perf. (%)	Sensitivity (%)	Specificity (%)
PD-HC (38)	47.4	50	45
MSA-HC (39)	71.8	83.3	61.9
PSP-HC (35)	80.0	77.8	82.4

**Table 2:** Classifier performance with preselection of features (patients versus healthy controls, number of cases in brackets). The percentage of principal components is arranged in order of highest to lowest variance accounted for and best number of PCs according to AIC. Highest performances are in bold.

%/number of PCs	In order of amount of variance					According to AIC		
	3%	5%	50%	70%	100%	1	3	5
PD-HC (38)	55.3	**63.2**	57.9	**63.2**	47.4	**63.2**	50	47.4
MSA-HC (39)	71.8	**74.4**	69.2	71.8	71.8	66.7	69.2	**74.4**
PSP-HC (35)	**82.9**	80	77.1	77.1	80	**82.9**	80	80

**Table 3:** Performance for binary classification of disease groups in the LOOCV. The number of cases per group is in brackets. The column Perf. indicates the percentage of subject cases correctly classified (all features included), Sensitivity indicates the percentage of correctly classified first disease group, Specificity indicates the percentage of correctly classified second disease group, and Perf. (AIC-5) indicates the performance when features are reduced to the best 5 PCs according to AIC.

Group	Perf. (%)	Sensitivity	Specificity	Perf. (AIC-5) (%)
PD versus MSA (41)	73.2	70	76.2	78
PD versus PSP (37)	67.6	80	52.9	70.3
MSA versus PSP (38)	68.4	76.2	58.8	71.1

**Table 4:** Performance for binary classification of disease groups (number of cases in brackets) in the LOOCV with feature preselection. The columns Feat. and Perf. indicate the percentage of features used and the corresponding performance. The remaining columns show confusion matrices and class accuracies. The number of subjects correctly classified for each class is in bold.

Feat. %	Perf. %	Class	PD (20)	PSP (17)	MSA (21)
100	65.5	PD	**15**	5	3
		PSP	4	**8**	3
		MSA	1	4	**15**
		Accuracy	75	47.1	71.4

50	67.2	PD	**15**	5	2
		PSP	4	**9**	4
		MSA	1	3	**15**
		Accuracy	75	52.9	71.4

25	69	PD	**15**	5	2
		PSP	4	**9**	3
		MSA	1	3	**16**
		Accuracy	75	52.9	76.2

**Table 5:** The LOOCV performance for various types of classifier. Features used were the subject scores obtained after applying the SSM/PCA method on all subjects included in the datasets. (∗) Note that for LDA only 90% of the features were considered because of the classifier's restrictions while constructing the covariance matrix. For easy reference, the feature preselection results for C4.5 already presented in Table 2 are included.

Dataset	PD-HC	MSA-HC	PSP-HC
Nearest neighbors	76.3	76.9	80.0
Linear SVM	78.9	92.3	88.6
Random forest	63.2	61.5	71.4
Naive Bayes	65.8	71.8	71.4
LDA (∗)	50.0	61.5	65.7
CART	57.9	53.8	85.7
C4.5	63.2	74.4	82.9
